# Behavioral and Brain Functions. A new journal

**DOI:** 10.1186/1744-9081-1-1

**Published:** 2005-04-22

**Authors:** Terje Sagvolden

**Affiliations:** 1 Editor-in-Chief, Department of Physiology, Institute of Basic Medical Sciences, University of Oslo, Oslo, Norway

## Abstract

*Behavioral and Brain Functions *(*BBF*) is an Open Access, peer-reviewed, online journal considering original research, review, and modeling articles in all aspects of neurobiology or behavior, favoring research that relates to both domains. *Behavioral and Brain Functions *is published by BioMed Central. The greatest challenge for empirical science is to understand human behavior; how human behavior arises from the myriad functions such as attention, language, memory and emotion; how these functions are reflected in brain structures and functions; and how the brain and behavior are altered in disease. *Behavioral and Brain Functions *covers the entire area of behavioral and cognitive neuroscience – an area where animal studies traditionally play a prominent role. *Behavioral and Brain Functions *is published online, allowing unlimited space for figures, extensive datasets to allow readers to study the data for themselves, and moving pictures, which are important qualities assisting communication in modern science.

## Introduction

*Behavioral and Brain Functions *(*BBF*) is an Open Access, peer-reviewed, online journal considering original research, review, and modeling articles in all aspects of neurobiology or behavior, favoring research that relates to both domains. *BBF *is published by BioMed Central.

The greatest challenge for empirical science is to understand human behavior, how human behavior arises from the myriad functions such as attention, language, memory and emotion, how these functions are reflected in the human brain, and how brain functions and behavior are altered in disease. Behavioral and cognitive neuroscience investigates the psychological, computational, and neuroscientific bases of normal and abnormal behavior. It is a field that receives a lot of attention through the *Brain Awareness Week *in March every year. The "*Decade of the Brain*" (1990–2000) was also important for promoting the field. The interdisciplinary nature of the field covers developments in human and animal behavioral science, neuroscience, neuropsychology, cognitive psychology, neurobiology, linguistics, computer science, and philosophy.

*Behavioral and Brain Functions *is the first Open Access journal for basic research covering the entire area of behavioral and cognitive neuroscience – an area where animal studies traditionally play a prominent role. *Behavioral and Brain Functions *is published online, allowing unlimited space for figures, extensive datasets to allow readers to study the data for themselves, and moving pictures, which are important qualities assisting communication in modern science.

## Open Access

*Behavioral and Brain Functions*' Open Access policy changes the way in which articles are published. First, all articles become freely and universally accessible online, and so an author's work can be read by anyone at no cost. Second, the authors hold copyright for their work and grant anyone the right to reproduce and disseminate the article, provided that it is correctly cited and no errors are introduced [1]. Third, a copy of the full text of each Open Access article is permanently archived in an online repository separate from the journal. *Behavioral and Brain Functions*' articles are archived in PubMed Central [2], the US National Library of Medicine's full-text repository of life science literature, and also in repositories at the University of Potsdam [3] in Germany, at INIST [4] in France and in e-Depot [5], the National Library of the Netherlands' digital archive of all electronic publications.

Open Access has four broad benefits for science and the general public. First, authors are assured that their work is disseminated to the widest possible audience, given that there are no barriers to access their work. This is accentuated by the authors being free to reproduce and distribute their work, for example by placing it on their institution's website. It has been suggested that free online articles are more highly cited because of their easier availability [6]. Second, the information available to researchers will not be limited by their library's budget, and the widespread availability of articles will enhance literature searching [7]. Third, the results of publicly funded research will be accessible to all taxpayers and not just those with access to a library with a subscription. As such, Open Access could help to increase public interest in, and support of, research. Public accessibility may become a legal requirement [8]. Fourth, a country's economy will not influence its scientists' ability to access articles because resource-poor countries (and institutions) will be able to read the same material as wealthier ones (although creating access to the internet is another matter [9]).

## Editorial Board

The editorial board covers a broad cross-section of brain and behavior research. Several members of the *BBF *Editorial Board are actively involved in promoting research and assisting researchers in poor and developing countries through *The International Brain Research Organization *(*IBRO*).

## Peer review policy

Publication of research articles in the broad area of behavioral and cognitive neuroscience is dependent on: relevance to the aims of the journal; scientific validity and excellence; and coherence with the research area as a whole, as judged by two independent reviewers. An Editorial Board oversees the peer review of manuscripts submitted to *Behavioral and Brain Functions*. Peer review is anonymous. A recommendation of acceptance or rejection is at the discretion of the Editors based on the reviewers' recommendations. Articles will be published immediately upon acceptance and soon after they will be listed in PubMed and archived in PubMed Central as well as other national archives.

## Competing interests

The Editor-in-Chief and the Editorial Board of *Behavioral and Brain Functions *have no financial incentive to accept manuscripts as they are not paid on the basis of the number of manuscripts accepted. In fact, they do not receive any financial remuneration for their involvement with the journal. We insist that decisions about a manuscript are based on the quality of the work, not on whether the authors' can pay the article-processing charge. Authors will be asked to declare any competing interests that they have.
